# The Evolution of Three Schizothoracinae Species from Two Major River Systems in Northwest China Based on Otolith Morphology and Skeletal Structure

**DOI:** 10.3390/biology13070517

**Published:** 2024-07-11

**Authors:** Chengxin Wang, Linghui Hu, Yong Song, Haoyang Xie, Liting Yang, Gulden Serekbol, Bin Huo, Shengao Chen

**Affiliations:** 1 College of Life Science and Technology, Tarim Research Center of Rare Fishes, Tarim University, Alar 843300, China; 10757203067@stumail.taru.edu.cn (C.W.); 10757222068@stumail.taru.edu.cn (L.H.); songyong@taru.edu.cn (Y.S.); haoyangxie2001@gmail.com (H.X.); 10757231127@stumail.taru.edu.cn (L.Y.); 2 College of Animal Science and Technology, Tarim Research Center of Rare Fishes, Tarim University, Alar 843300, China; 10757231098@stumail.taru.edu.cn; 3 College of Fisheries, Huazhong Agricultural University, Wuhan 430070, China; huobin@mail.hzau.edu.cn

**Keywords:** evolution, Schizothoracinae, otolith morphology, skeletal structure

## Abstract

**Simple Summary:**

Based on the differences in otolith morphology and skeletal morphology, this study presents an investigation of the evolution of three Schizothoracinae species from two major river systems in Northwest China. A total of 138 samples were collected, and the morphological factors, morphological indices and Fourier transform coefficients of the otoliths were measured and analyzed to compare the morphological differences between the otoliths and bones. Morphological analysis of the otoliths of the three Schizothoracinae species revealed significant differences and cluster analysis further confirmed this relationship. At the same time, their skeletal morphology showed significant differences in the number of tentacles, pharyngeal teeth and vertebrae, as well as in the shape of several key bone blocks. The unique morphological characteristics and skeletal differences of the three Schizothoracinae species during their evolution are closely related to the geological changes in and water system evolution of the Qinghai-Tibet Plateau. This study not only deepens our understanding of the evolution of plateau fishes and other species but also provides a scientific basis for understanding the conservation and evolutionary mechanism of plateau fishes, which is highly important for ecological protection and biodiversity conservation.

**Abstract:**

Schizothoracinae species are the largest group of Cypriniformes that readily adapt to the natural conditions of the Qinghai-Tibet Plateau. This group has habitat characteristics and distribution patterns centered on the Qinghai-Tibet Plateau. To study the evolution of three Schizothoracinae species in Northwest China, the evolutionary characteristics of these species were explored based on differences in otolith morphology and skeletal morphology. From 2020 to 2022, 138 samples (63 *Aspiorhynchus laticeps*, 35 *Diptychus maculatus* and 40 *Schizothorax pseudaksaiensis*) were collected from the Tarim River and Ili River, 6 basic morphological parameters of otoliths were measured and converted into 6 morphological factors and 7 morphological indices. A total of 77 Fourier transform coefficients of each otolith were selected The first three principal components accounted for 92.834% of the total variation in 13 otolith morphological indices of the three Schizothoracinae species, and the overall discrimination rate was 94.20%. According to the principal component analysis of 77 Fourier harmonic values of otoliths, the first 20 principal components explained 97.233% of the total variation, and the overall discrimination rate was 100%. The results of the cluster analysis directly reflected the relationships between related species. The differences in the bone morphology of the three Schizothoracinae species were particularly reflected in the number of whiskers, pharyngeal teeth and vertebrae, and there were also significant differences in the shapes of the sphenotic (SP), pterotic (PTE), preoperculum (PO), branchiostegal ray (BRA) and basibranchial (BB) bones. Their unique morphological and skeletal characteristics are closely related to geological changes and water system evolutionary trends. This study contributes to the understanding of species identification and the evolutionary status of plateau fishes, provides a reference for further evolutionary classification and for assessing the evolutionary mechanisms of plateau fishes, and provides a scientific basis for phylogeny and germplasm resource protection.

## 1. Introduction

The morphological characteristics of organisms differ in different environments, which is usually the result of long-term adaptation to the living environment [1]. For aquatic animals, otolith morphology has been widely used to identify fish in marine and freshwater ecosystems, and such studies have focused mainly on changes in otolith morphology. Otoliths are calcium carbonate condensate in the inner ear of fish and are considered unique among vertebrates and their form is influenced by biological and abiotic factors [2]. Biological aspects are controlled by heredity [3], while nonbiological aspects are affected by the habitat environment [4]. The combination of these two factors determines the overall morphology and growth pattern of otoliths. Otolith morphology is influenced by individual development and habitat and is often used in comparative studies between different species [5,6]. Berg et al. [7] emphasized the strong influence of genetic factors, while morphological variability is also influenced by exogenous factors such as temperature, depth, salinity, substrate and feeding conditions [8,9,10,11]. Studies investigated the otolith morphology and growth of *Dosidicus gigas* in different waters of the Eastern Pacific Ocean [12] and the morphological characteristics and differences of *Saurogobio dabryi* otoliths in different basins of the Jialing River [13]. These studies have attempted to explain the relationship between otolith morphology and environmental adaptation. In specific water environment conditions, the otolith morphological characteristics of the same fish species are highly homogeneous due to the same environmental conditions and habitat characteristics [14], while in different water environments, to better adapt to different water temperatures, water flow speeds and other environmental factors, otolith morphological characteristics are heterogeneous [15]. Therefore, otoliths are effective markers for studying the ecological adaptability of fish. It is important for understanding and delineating the ecological habits, population dynamics and evolutionary history of recent and extinct species. It can be used to study changes caused by environmental factors and to understand the influence of different habitats on morphological changes in otoliths [16].

The skeletal system is an important tissue that supports the body, protects internal organs and coordinates muscle movement in fish. In the long-term evolutionary process, the development sequence, shape and structure of various skeletons have undergone remarkable adaptations to their habitat environment. The morphological and structural characteristics of the skeletal system of fish are important for studying the evolution and classification of fish systems [17] and are also important for further studying the functional development of fish tissues and organs [18]. Studies on *Dentexdentex*, *Alosas apidissima* and *Trachinotus ovatus* have shown that bony fishes differ significantly in bone development due to species differences [18,19,20]. Bone supports and protects the fish, and the study of structural differences is the main external manifestation of adapting to different environments, which is of great significance for the morphology and taxonomic evolution of fish [21,22,23].

There are more than 100 species of Schizothoracinae in 15 genera worldwide, and 90 species (subspecies) in 12 genera are found in China [24,25,26]. They are the primitive barbodyae fishes distributed in Qinghai–Tibet in the Late Third Age, and the only natural group of Cyprinidae adapted to the special environment of the plateau [27]. As a reference for the uplift and crustal changes in the Qinghai-Tibet Plateau, Schizothoracinae species are divided into three groups, namely, primitive, specialized and highly specialized, according to the number of whiskers, the number of hypopharyngeal tooth rows and body scales resulting from differences in habitat elevation. Xinjiang has two groups, primitive and specialized [28,29,30]. Research on this group is mainly based on aspects such as classification, individual ecology and phylogeny. Different species in this group lived in different habitats at different altitudes, and the vertical and horizontal spatial distribution of living waters were also different, showing obvious environmental heterogeneity. *Aspiorhynchus laticeps* (Day 1877) from the Tarim River system, *Diptychus maculatus* (Steindachner 1866) from the Taxkorgan River and *Schizothorax pseudaksaiensis* (Racoma) (Herzenstein 1889) from the Ili River system belong to Schizothoracinae but belong to different levels of schizothorax specialization. After the late Tertiary period, the Qinghai–Tibet Plateau experienced three sharp rises and uplifts, and the crustal changes resulted in different elevations and environmental conditions in the inhabited areas, which resulted in the formation of different genera of fish [31]. Schizothoracinae is one of the most important taxa in the Tarim Basin and its surrounding mountains. Its origin and evolution may be closely related to the uplift of the Kunlun, South and North Tianshan Mountains and to geological and climatic changes in the Tarim Basin. The differences in otolith morphology and bone structure of Schizothoracinae may reflect these changes at the biological evolution level. Therefore, through the in-depth study of these characteristics of Schizothoracinae, the influence of geological and watershed changes on their faunal evolution in the Tarim Basin and its surrounding areas are discussed, so as to accumulate theoretical data for the evolutionary classification and species protection of plateau fishes.

## 2. Materials and Methods

### 2.1. Sample Collection

**Figure 1:**
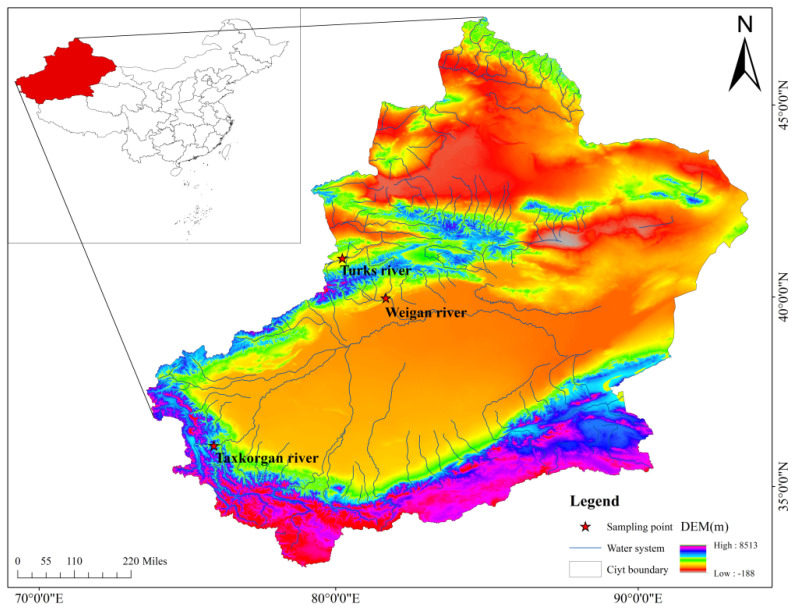
The sampling area of three Schizothoracinae species.

**Figure 2:**
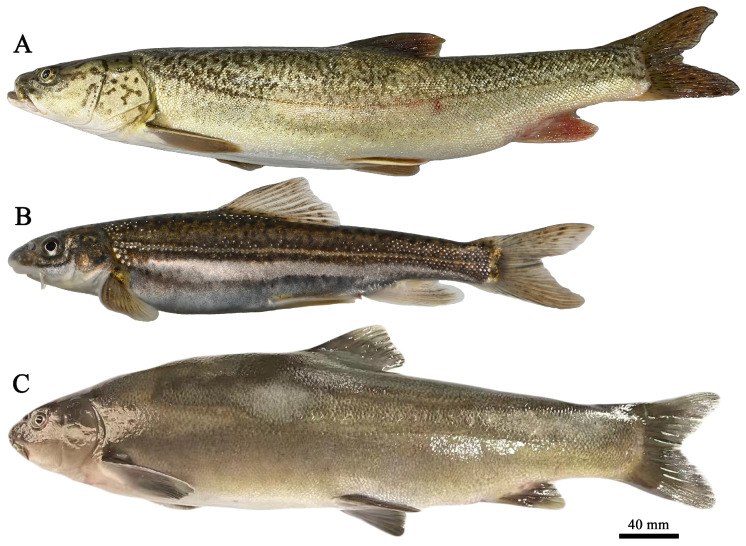
Images of three Schizothoracinae species: Note: (**A**). *Aspiorhynchus laticeps*; (**B**). *Diptychus maculatus*; and (**C**). *Schizothorax pseudaksaiensis*.

This study was approved by the Animal Care and Use Committee of Tarim University (approval code: TDD-KYXF 20200426). From 2020 to 2022, a total of 138 samples of *A. laticeps* were collected from the Weigan River in the Tarim River system, *D. maculatus* from the Taxkorgan River system and *S. pseudaksaiensis* from the Turks River in the Ili River system (Figure 1) by means of floating gillnets, fixed gillnets and ground cages (with a mesh size of 2 cm). The three Schizothoracinae species are shown in Figure 2. Basic biological information of the fish samples was measured immediately after they were caught (e.g., standard length/mm, body weight/g, etc.); 5 fish were selected for bone anatomy analysis. All of them were sexually mature individuals with normal body size, no deformities and no apparent damage, and all fins were intact. For detailed information, see Table 1. The lapillus otoliths were extracted, and the otolith envelope and mucus were removed, cleaned and stored in a centrifuge tube containing 95% ethanol. The otoliths used in this study were all microotoliths, hereinafter referred to as otoliths.

The reagents and equipment used were 4% H_2_O_2_ (Tianjin Beilian Fine Chemicals Development Co., Ltd., Tianjin, China), 3% NaOH (Tianjin Yongsheng Fine Chemical Co., Ltd., Tianjin, China), C_8_H_10_ (Tianjin Zhiyuan Chemical Reagent Co., Ltd., Tianjin, China), a 500 mL beaker, an electronic temperature control furnace (DL-1, Beijing Yongguang Medical Instrument Co., Ltd., Beijing, China), an electronic scale (BSM-3200.2, Shanghai Zhuojing Electronic Technology Co., Ltd., Shanghai, China), a camera (Powershot A 3000IS, Canon, China), a Micro CT μ80 micro CT (Shanghai Yinxi Biotechnology Co., Ltd., Shanghai, China), etc.

### 2.2. Otolith Measurement

To reduce data errors and maintain data accuracy, right otoliths were selected for measurement. Using a microscope (ACT-2, Nikon SMZ1270i; Shanghai, China), a camera was used to record a digitized image of the otolith against a black background. NIS-Elements 4.0 software was used to calibrate the otolith in both the horizontal and vertical directions, and 6 otolith parameters (mm) that characterize the morphology of the otolith were estimated: the otolith area (OA), minimum radius of otolith (R_min_), maximum radius of otolith (R_max_), otolith perimeter (OP), otolith length (OL) and otolith width (OW). In addition, various shape descriptors were calculated, namely, *F*_1_–*F*_6_, roundness (*R*o), the format factor (Ff), circularity (*C*), rectangularity (*R*e), ellipticity (*E*), the radius ratio (*R*r) and the aspect ratio (*A*r) [32,33] (Table 2).

SHAPE 1.3 software was used to extract elliptical Fourier descriptors (EFDs) from otolith images. The morphology of the otolith was decomposed into n Fourier harmonic values, and the differences between the figures were compared based on these harmonic values. During the reconstruction and transformation of the overall otolith morphological contour, morphological information was extracted. In the transformation of 20 harmonic degrees, each harmonic degree had 4 coefficients, which were represented by a, b, c and d [34,35]. When the EFD coefficients were normalized, the first 3 EFD coefficients in the first harmonic order were set to a fixed value (a1 = 1, b1 = c1 = 0) so that the overall morphological profile information of each otolith was actually expressed by 77 EFD coefficients.

### 2.3. Bone Preparation

CT scanning [36]: The whole fish samples (n = 5) were analyzed with a Micro CT-μ80 micro CT (BRUKER SKYSCAN 1276). Scanning conditions: voltage (70 kVp), current (114 μA), 360° rotational scanning, scanning 180 min, resolution 14 um, average frame 4, angle gain 0.4°. A total of 500 images of 2 048 × 048 pixels with different cross-sections of the same sample were scanned, and the skeletal system was reconstructed using μCT Ray v4.0-1.

Boiling-based meat removal [37]: Six steps were used to prepare the bone of the specimens (n = 5), including boiling, meat removal, etching, degreasing, bleaching and dehydration.

Individual bone specimens were observed with an anatomical microscope (SMZ-140 N2GG, MOTIC, Xiamen) and photographed (PowerShot A3000 IS, Canon, Zhuhai, China). Comparative analysis was performed after processing with Photoshop CS6.

### 2.4. Data Analysis

#### 2.4.1. Standardized Otolith Measurement Parameters

The raw data from 138 samples were examined for outliers that could indicate errors or specimen damage, and the body length distributions of the three Schizothoracinae species were compared. The size distribution (*SL*) of the three Schizothoracinae species varied (Table 1). This is undesirable because differences between the otolith samples may be partly due to differences in the size of the fish. To remove the dimensional component from shape measurements, all individual morphometric feature measurements were standardized according to the following formula [38]:

*M*s = *M*o(*L*s/*L*o)*^b^*

where *M*s is the standardized measurement value; *M*o is the measured characteristic length; *L*s is the population (arithmetic)-averaged standard length of all fish in all samples in each analysis; *L*o is the standard length of the sample; and *b* is estimated by the allometric growth equation *W* = a*L*^b^ for each trait based on the observed data.

#### 2.4.2. Principal Component Analysis

Through ORIGIN 9.0 analysis, several principal components were obtained from 13 otolith morphological indices and 77 EFD coefficients were extracted based on otolith contours. The eigenvalues, variance contribution rates and cumulative variance contribution rates of these principal components were calculated, and two-dimensional and three-dimensional scatter plots were constructed for principal component analysis to determine the traits that ultimately affect the otolith morphology of the three Schizothoracinae species.

#### 2.4.3. Discrimination Analysis

According to the known otolith morphology data, Wilks’ Lambda method selects the parameter that contributes the most to the discriminant and establishes the discriminant function. The sample will be classified into the corresponding category of the function with the larger Y value obtained. The classification of three Schizothoracinae species was determined by SPSS 18.0. The formula of discriminant accuracy refers to the method of Li Sifa et al. [39]. 

#### 2.4.4. Cluster Analysis

Through SPSS 18.0 analysis, the average values of the otolith morphology data of the three Schizothoracinae species were calculated, the European systematic clustering method was subsequently used to cluster these values, and the degree of affinity between them was determined via a tree diagram.

## 3. Results

### 3.1. Otolith Morphology

#### 3.1.1. Otolith Morphological Characteristics

The size of the otoliths was small (Table 3). The Shapiro-Wilk test revealed that the otolith area, minimum radius of the otolith, maximum radius of the otolith, otolith perimeter, otolith length and otolith width did not conform to the normal distribution (Table 3).

#### 3.1.2. Nonparametric ANOVA

Nonparametric analysis of variance based on the Kruskal-Wallis test revealed that 13 otolith morphological indices of the three Schizothoracinae species were significantly different (*p* < 0.05) (Table 4). Postmortem tests based on the Mann-Whitney U test further revealed significant differences in 13 otolith morphological indices (*p* < 0.01).

#### 3.1.3. Principal Component Analysis

**Figure 3:**
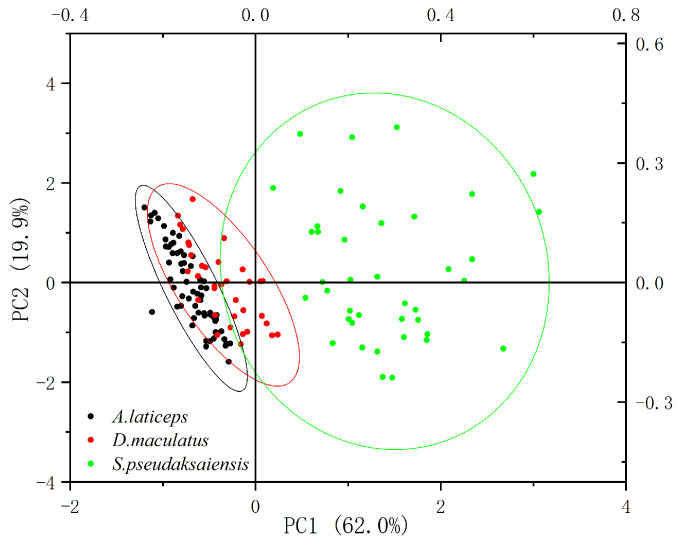
Two-dimensional scatter plots of the first and second principal components of 13 morphological indices of the otoliths of three Schizothoracinae species.

**Figure 4:**
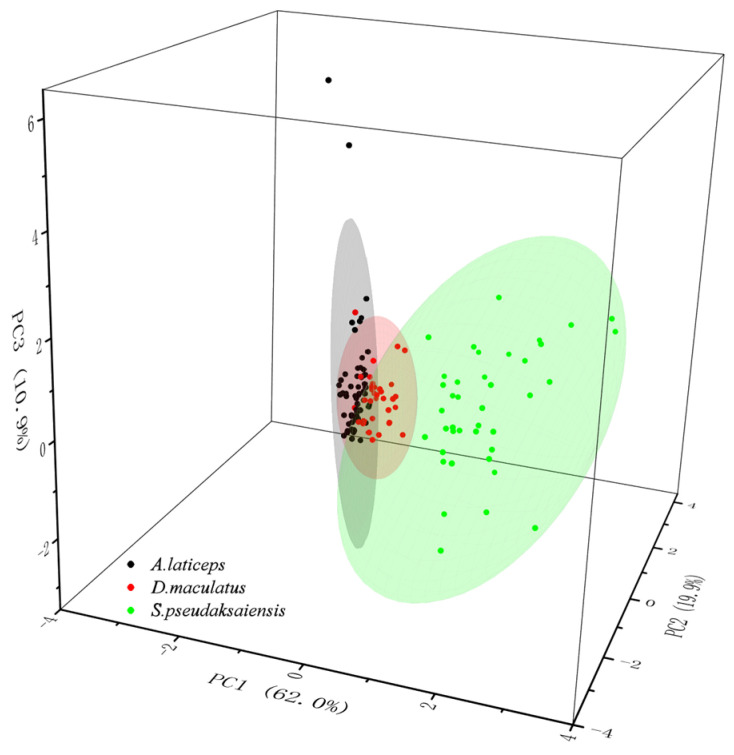
Three-dimensional scatter plots of the first three principal components of 13 morphological indices of the otoliths of three Schizothoracinae species.

According to the KMO statistics (KMO = 0.634) and Bartlett’s sphericity test (*p* < 0.01), there was a significant correlation between species variables, indicating that the morphological differences of otolith among the three Schizothoracinae species were suitable for principal component analysis. Both 2D and 3D scatter plots show that the three Schizothoracinae species have good differentiation in the first 2/3 principal components selected (Figure 3 and Figure 4). The contribution rates of the first three principal components were 62.022%, 19.903% and 10.905%, respectively, and the cumulative contribution rate was 92.834% (Table A1). The first principal components were *F*_4_, *F*_5_, *R*o and *F*f, which mainly reflect the relationship between the length and breadth of the otolith axis. The second principal components were *F*_6_, *R*o and *E*, which mainly reflect the regularity and roundness of the otolith contour. The third principal components were *F*_2_, *F*_3_ and *R*e, which mainly reflect the thickness of the otolith and its relationship with the rectangle.

**Figure 5:**
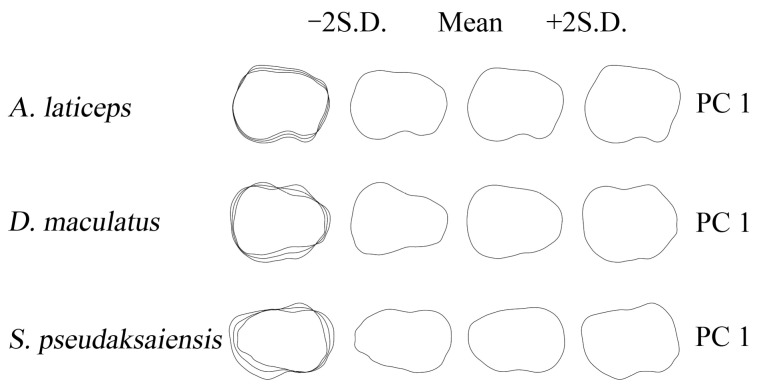
Outer contour of the otoliths of three Schizothoracinae species.

The principal component analysis results showed (Table A2) that the cumulative contribution rate of the first 20 principal components of the parameter values of the three Schizothorax species was 97.233%. The first 3 principal components explained 61.582% of the total variation, and the first 10 principal components explained 89.7000% of the total variation. A contour reconstruction of the effective principal components is shown in Figure 5.

#### 3.1.4. Discrimination Analysis

Five otolith morphological indices (*F*_2_, *F*_3_, *F*_5_, *F*_6_ and *Rr*) and eight parameters (A17, A20, B15, C13, C15, C17, C20 and D13) that contributed substantially to the discrimination were selected. Instead of *X*_1–5_ and *Z*_1–8_, the specific discrimination formulas were as follows:

Discrimination based on 13 otolith morphological indices:

*Y*_A_ = −112,939.492 − 31,768.494*X*_1_ + 25,073.961*X*_2_ − 1229.621*X*_3_ + 293,057.033*X*_4_ + 3868.712*X*_5_;

*Y*_D_ = −113,065.184 − 31,783.111*X*_1_ + 25,087.655*X*_2_ − 1234.657*X*_3_ + 293,232.405*X*_4_ + 3888.990*X*_5_;

*Y*_S_ = −112,989.617 − 31,777.853*X*_1_ + 25,074.877*X*_2_ − 1236.204*X*_3_ + 293,005.781*X*_4_ + 3952.753*X*_5_.

Discrimination based on 77 Fourier harmonic values:

*Y*_A_ = −1042.063 − 12,731.893Z_1_ + 10,860.016Z_2_ + 17,905.530Z_3_ − 14,982.815Z_4_ + 13,722.521Z_5_ − 13,621.140Z_6_ − 33,364.152Z_7_ − 26,144.604Z_8_;

*Y*_D_ = −996.936 − 13,088.797Z_1_ + 11,685.204Z_2_ + 17,101.718Z_3_ − 16,688.436Z_4_ + 13,214.442Z_5_ − 16,327.820Z_6_ − 28,762.691Z_7_ − 22,007.676Z_8_;

*Y*_S_ = −877.963 − 12,062.675Z_1_ + 11,727.743Z_2_ + 12,418.435Z_3_ − 20,257.699Z_4_ + 9628.468Z_5_ − 17,131.149Z_6_ − 28,020.568Z_7_ − 20,189.952Z_8_.

**Figure 6:**
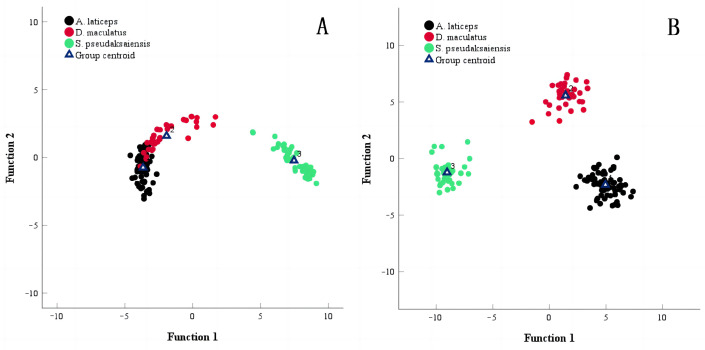
Scatter plot of scores based on the first two canonical discriminant functions. Note: (**A**). based on 13 otolith morphological indices; and (**B**). based on 77 Fourier harmonic values.

The results showed that among 138 individuals of three Schizothoracinae species, 4 out of 63 individuals of *A. laticeps* were misjudged and the success rate was 93.70%. Among 35 individuals of *D. maculatus*, 4 were misjudged and the success rate was 88.60%. Among 40 *S. pseudaksaiensis* individuals, 0 were misjudged and the success rate was 100.00%. The overall success rate was 94.20% (Table 5). Based on the discrimination formula of 77 Fourier harmonic values, there were 0 misjudgments in Platyrostris and the success rate of discrimination was 100%. The discrimination results were the same for the other two Schizothoracinae species (Table 5). Scatterplots of the results of the typical discrimination analysis for the three Schizothoracinae species were obtained via stepwise discrimination analysis (Figure 6).

#### 3.1.5. Cluster Analysis

**Figure 7:**
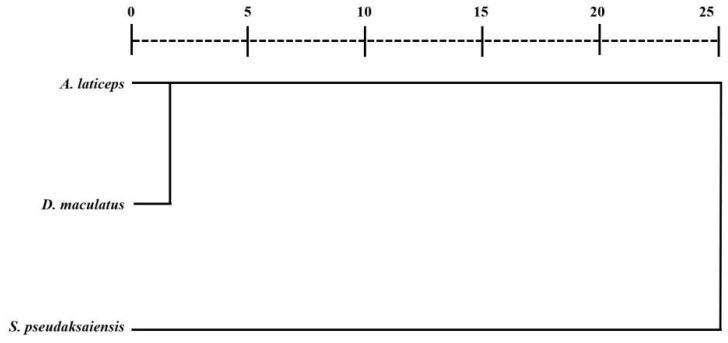
Systematic clustering of three Schizothoracinae species based on the class average method.

Clustering analysis revealed that the Euclidean distance between *A. laticeps* and *D. maculatus* was the smallest, indicating that they were the closest genetically and closely related in evolutionary history. The Euclidean distance between *S. pseudaksaiensis* was greater than that between the other two Schizothoracinae species, and the relatives are more distant (Figure 7, Table A3).

### 3.2. Skeletal Anatomy

#### 3.2.1. Cranial Anatomical Observation

**Figure 8:**
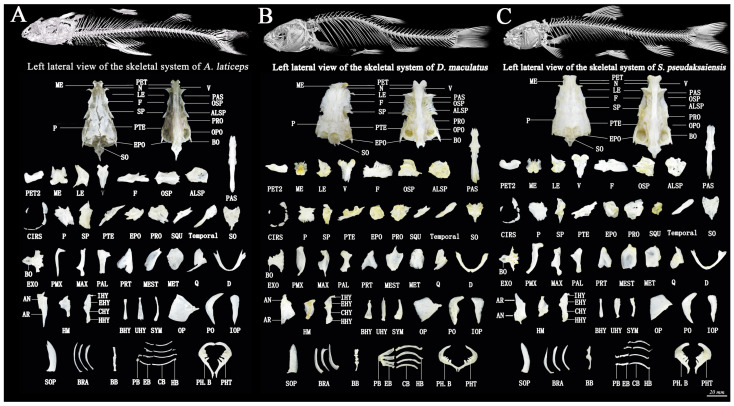
Skeletal system of the cranial and pharyngeal skulls of three Schizothoracinae species: (**A**). *Aspiorhynchus laticeps*; (**B**). *Diptychus maculatus*; and (**C**). *Schizothorax pseudaksaiensis*. Note: PET: Preethmoid; PET2: 2nd preethmoid; ME: Mesoethmoid; N: Nasal bone; LE: Lateral ethmoid; V: Vomer; F: Frontal bone; PAS: Parasphenoid; OSP: Orbitosphenoid; SP: Sphenotic; ALSP: Alisphenoid; P: Parietal bone; PTE: Pterotic bone; PRO: Prootic; EPO: Epiotic; SO: Supraoccipital; BO: Basioccipital; EXO: Exoccipital bone; SQU: Squamosum; Temporal: Temporal bone; PMX: Premaxilla; MAX: Maxilla; PAL: Palatine bone; PRT: Pterygoid; MEST: Mestapterygoid; MET: Metapterygoid; Q: Quadratebone; D: Dentary; AR: Articular bone; AN: Angulare; IHY: Interhyoid; EHY: Epihyoid; CHY: Ceratohyal; HHY: Hypohyoal bone; HM: Hyomandibular bone; BHY: Basihyoid; UHY: Urohyoid; SYM: Symplectic bone; L: Lacrimal bone; CIRS: Circumorblital series; OP: Operculum; PO: Preoperculum; IOP: Interoperculum; SOP: Suboperculum; BRA: Branchiostegal ray; PB: Pharyn-gobranchial; EB: Epibranchial; CB: Ceratobranchial; HB: Hypobranchial; BB: Basibranchial; PH.B: Pharyngeal bone; PHT: Pharyngeal tooth.

The skulls of the three Schizothoracinae species are long, and the skulls of *A. laticeps* are slenderer and more than twice as long as the widest parts. *D. maculatus* and *S. pseudaksaiensis* have wide crania, approximately twice as long as the widest cranium, with a short snout and a short head region behind the eyes (Figure 8).

The second preethmoid (PET2) of *A. laticeps* is concave inwards in a “√” shape and has high curvature, and both ends are rounded and prominent. The lateral ethmoid (LE) and vomer (V) of *A. laticeps* are significantly different from those of the other two Schizothoracinae species. The PET2 of *D. maculatus* is rod-shaped, but one end extends outwards in a pointed convex shape. The lateral edge of the LE is not significantly depressed, the front end of V is wide, the back end is pointed and the front end of the depression is obtuse. The nasal bone (N) of *S. pseudaksaiensis* is not boot-like; the LE border depression was not significant. The frontal bone (F) of *A. laticeps* is more elongated than that of the other Schizothoracinae species. The F layers of *D. maculatus* and *S. pseudaksaiensis* are knife-shaped, and the bone slices are wider. The alisphenoid (ALSP) layers of the three Schizothoracinae species are slender, equal in the left and right parts and long and narrow in the back.

The sphenotic (SP) and pterotic bone (PTE) of the three Schizothoracinae species are significantly different, and the upper end (SP) of *D. maculatus* is slightly concave; the two ridges on one side of the wing ear bone are more obvious than those of the other two Schizothoracinae species, and the two ends of the PTE of *D. maculatus* extend outwards in a sharp protrusion.

#### 3.2.2. Pharyngocranial Anatomy Observation

There were significant differences in the premaxilla (PMX), maxilla (MAX) and mestapterygoid (MEST) indices among the three Schizothoracinae species. *D. maculatus* has an obvious curved hook at one end of the PMX and a sharp protrusion at one end of the MAX. *S. pseudaksaiensis* has a gentle curvature at the top of the PMX and a slight curvature at the bottom; the palatine bones (PALs) are thick and blunt; the pterygia (PRTs) are roughly symmetrical; and the MESTs are slightly teardrop shaped. One end of the hyomandibular bone (HM) of *A. laticeps* is slenderer than that of other Schizothoracinae species, and the whole symplectic bone (SYM) is slenderer than that of other Schizothoracinae species

The preoperculum (PO), three pairs of branchiostegal rays (BRA) and three pieces of the basibranchial (BB) of *A. laticeps* are more elongated than those of the other two Schizothoracinae species. The teeth of *D. maculatus* are 3·4/4·3, with two rows of pharyngeal teeth (PHT) on the inside. The teeth of *A. laticeps* and *S. pseudaksaiensis* are 2·3·5/5·3·2, with three rows of pharyngeal teeth (PHT) on the inside. The appearance and number of pharyngeal bones and teeth of *D. maculatus* punctatus are significantly different from those of the other two Schizothoracinae species.

#### 3.2.3. Trunk and Tail Anatomy

The number of Platyrostris vertebrae is 4 + 40–41 + 1, and there are ribs from the 5th vertebra to the 16th or 17th vertebra between 21 and 22. The number of Platyrostris vertebrae is 4 + 34–35 + 1, and there are ribs from the 5th vertebra to the 22nd or 23rd vertebra between 22 and 23. The number of vertebrae of Schizothorax yiliensis is 4 + 44–45 + 1, and there are ribs between the 5th and 28th or 29th cones, ranging from 23 to 24 (Figure 8). The odd fin bones of these three Schizothoracinae species are compared as follows (Table 6).

## 4. Discussion

The differences in otolith morphology among different fish species are mainly caused by genetic factors and are influenced by their external living environment [40,41]. According to the results of the otolith morphology study, the morphological data of six otoliths (OA, R_max_, R_min_, OP, OL and OW) among the three Schizothoracinae species showed significant differences (*p* < 0.01), indicating that these fishes had significant differences in otolith morphology. *S. pseudaksaiensis* and *A. laticeps* are distributed at altitudes of 500–1000 m, while *D. maculatus* is distributed at altitudes of 1000–1500 m or more [31]. The elevations of Lop Nur (768 m) and the main stream of the Tarim River (800~900 m) are suitable for *A. laticeps*, suggesting that the different living habits of these species may also be responsible for the differences. In addition, the shape of the fish also has a certain impact on the shape of the otoliths. *A. laticeps* and *D. maculatus* migrate to specific waters to spawn every breeding season [31], which may lead to adaptive changes in the shape of the fish, thus affecting the overall shape of the otoliths [42]. The morphological characteristics of the three Schizothoracinae species may be the result of the combined effects of genetic factors and the living environment.

Otolith morphology is highly species-specific and group-specific, and the extraction of otolith morphological features can not only allow the age determination but also be an effective means of species classification and identification [43]. A high identification success rate has been achieved in many empirical studies on fish species based on otolith morphology [44,45]. In this study, the morphological changes in the otoliths of three Schizothoracinae species were accurately analyzed. Compared with the otolith morphological indices, the elliptical Fourier coefficient had a better overall identification rate for Schizothoracinae species, which is consistent with different genera of fish [46,47,48,49], confirming that otolith morphology is related to genetic properties and can be used to identify fish species. According to the scatterplot of the typical discrimination function (Figure 6), the three Schizothoracinae species have large group centroid distances and obvious differences in their distribution, which can be easily distinguished, and the discrimination effect is ideal. The clustering relationship was consistent with the results of the principal component analysis and discrimination analysis, which directly reflects the species relationship. The evolutionary distance between *A. laticeps* and *D. maculatus* was the smallest, and the kinship was the closest, followed by *S. pseudaksaiensis*, indicating that the otolith morphology of these three Schizothoracinae species is related to genetic properties. This is consistent with the results of the cluster analysis on the morphological characteristics of the otolith of Carangidae species by Ou et al. [50], which can clearly see the process of cluster change, as well as the relationships between species and the change in morphological differences. There is good potential for classifying fish species and carrying out cluster analysis based on otolith morphology in Schizothoracinae because of the remarkable specificity of otolith morphology in different species.

The growth and development of fish bones are closely related to genetic, nutritional and environmental factors [51]. Skeletal morphological structure is relatively stable, and its morphological characteristics are an important basis for identification and classification [52]. The results of this study showed that the three Schizothoracinae species could be distinguished based on the bone block shapes of the SP, PTE, PO, BRA and BB. The uplift of the northern margin of the Qinghai-Tibet Plateau blocked the Indian Ocean summer monsoon and strengthened the Siberian winter monsoon, which jointly accelerated the aridity of the Tarim Basin [53,54,55]. The climatic environment of the Tarim Basin has been transitioning towards increasing dry heat since the late Cretaceous [56]. The arid environment increased the salinity of the seawater remaining during the process of retrogression in this area, and the fissure fish in this area gradually adapted to the rivers and lakes formed by the uplift of the West Kunlun Mountains [57] and the North Tianshan Mountains [58]. An aeolian sand environment [59] salinizes or dries Haigi Lake in the basin, forming a natural isolation area for Schizothoracinae between the North Tianshan Mountains and the West Kunlun Mountains. The West Kunlun Mountains may be the center of origin of *D. maculatus*, and the North Tianshan Mountains may be the center of origin of *S. pseudaksaiensis*. More extensive and long-term species exchange and diffusion occurred between the Tarim Basin and the surrounding mountain water systems and among tributaries in the basin [60]. The southern Tianshan uplift [61] blocked the dispersal of Schizothoracinae from the Ili Valley to the Tarim Basin.

In terms of elevation, as the elevation distributions of the three Schizothoracinae species previously studied are different, *A. laticeps* has developed a morphological trait with one pair of whiskers [62,63]. The whiskers of *D. maculatus* were reduced to one pair. The uplifted South Tianshan [61] connects the North Tianshan and the West Kunlun Mountains, and *D. maculatus* may have adapted to the high altitude and spread along the alpine rivers and lakes originating in the South Tianshan. To cope with extreme weather, fulfill reproductive needs and maintain normal life activities, high-altitude fish need to expand their diet and increase their rate of food intake to accumulate energy. When the number of whiskers is lower, the fish’s sensory functions weaken, and their dietary range broadens [27]. Further, there are three rows of hypopharyngeal teeth in *A. laticeps* and *S. pseudaksaiensis* and two rows in *D. maculatus*, and the decrease in the number of pharyngeal tooth rows is related to its more extensive omnivorous habit. The lateral pharyngeal teeth play a major role in feeding, but the grinding ability of the medial hypopharyngeal teeth (only two teeth) weakens or even disappears with an increasingly omnivorous diet [27]. Therefore, the variation in the number of rows of hypopharyngeal teeth further verified that *D. maculatus* is a more specialized class of Schizothoracinae. *A. laticeps* is a fierce fish that lives in slow-flowing waters. Its small number of vertebrae and ribs lends skeletal structural support to allow for explosive swimming capacity in pursuit of prey [64].

In the evolutionary adaptation of fish, their growth is closely related to water temperature and aquatic organisms [65,66], and they can obtain required nutrients by predation on aquatic organisms (such as plankton, benthos, aquatic plants, etc.). *A. laticeps* feeds on fish, is large, lives at relatively low altitudes [31], and may enter the Tarim Basin from the surrounding mountains via surface runoff. In this area, the flow of low-altitude rivers and tail lakes is slow (some still water is present), the basic productivity is high, and the abundance of prey organisms (including fish) is high. The flat snout fish that enter these waters gradually develop large sizes, mouth ends, mouth splits, etc., which are adapted for feeding [67]. In addition, the water supply on the plateau mainly depends on the melting of ice and snow, so the water temperature is very low, and diatoms adapted to the low-temperature environment are the main prey [68]. In the Taxkorgan River basin, river spotted dipterus are more widely distributed around the Taxkorgan Hydropower Station, which is rich in prey organisms [69], and filamentous algae, diatoms and organic debris are abundant in the whipstone, which enables the fish to have more flexible food choices and allows them to prey on different kinds of prey organisms in different seasons. This is further verified by the degradation of the hypopharyngeal teeth rows of *D. maculatus*. In addition, the unique hydrological, geographical and ecological environment of the region with clear river water, rocky and sandy bottoms, low sand content and relatively smooth water flow further promoted the distribution of *D. maculatus* in the basin [70]. In the Turks River, the biomass of phytoplankton in autumn is the highest (94.8% of diatom), 34 species of zooplankton, and many benthic animals, such as hookshrimp and midge larvae [71], which serve as effective food sources for *S. pseudaksaiensis*. In addition, the water flow varies greatly, and the water temperature is relatively low, with the highest water temperature being 17 °C [72,73,74] during the year. The uniform growth of *S. pseudaksaiensis* [62] indicates that it adapts well to environmental changes, and it may find suitable habitats when seasonal conditions change. The adaptability of the three Schizothoracinae species to the environment changed with water temperature during long-term evolution [65,66], and the fish growth trends were closely related to the aquatic organisms in their habitats.

The unique evolutionary history and ecological adaptability of plateau fishes play important roles in maintaining the stability and biodiversity of plateau ecosystems. The three Schizothoracinae species exhibit unique morphological evolution characteristics and skeletal differences, which are closely related to their ability to adapt to special plateau environments. Therefore, the ecological needs and habitat protection of these fish should be considered to avoid human interference, such as overfishing and environmental pollution. Conservation plans should be developed for the diversity of plateau fish species, including the establishment of protected areas, restrictions on fishing activities and pollution reduction measures to ensure that these fish species can thrive in their natural habitats. The aim of this study was to further protect the biodiversity of these fish, provide a scientific basis for population management and protection and maintain the stability and integrity of plateau ecosystems.

## 5. Conclusions

A high spatial heterogeneity of aquatic ecosystems on the Qinghai-Tibet Plateau has supported the rich diversity of Schizothoracinae. Schizothoracinae species adapted to survive in alpine water environments and high saline-alkali areas and exhibit unique skeletal character, with clearly differentiated otolith shapes in the three investigated Schizothoracinae species. There were significant differences among the skeletal systems of the three Schizothoracinae species, especially in terms of the number of whiskers, number of pharyngeal teeth and number of vertebrae, as well as in the shape and structure of the SP, PTE, PO, BRA and BB bones. The unique morphological and evolutionary characteristics and skeletal differences of these three Schizothoracinae species are closely related to the uplift of the Tibetan Plateau, and their faunal composition and spatial distribution patterns have gradually broadened in recent years due to the combined effects of multiple factors, such as selection pressure, interspecific and intraspecific competition and hydrological and climatic factors. This study advances our understanding of the origins, evolutionary processes and dynamics of Schizothoracinae species and serves as a reference for assessing the diversity of other Schizothoracinae species.

## Figures and Tables

**Table 1:** Sample information of three Schizothoracinae species.

	Species	Time	Site	Altitude	n	Standard Length/mm	Body Weight/g
						Mean ± S.D.	Mean ± S.D.
Otolith morphology sample	*Aspiorhynchus laticeps*	Jul. 2020 (Summer)	E82°27′12.9″, N41°44′59.2″	1110 m	63	4.71 ± 1.11	70.69 ± 5.84
	*Diptychus maculatus*	Aug. 2020 (Summer)	E75°56′27.5″, N37°49′54.4″	2183 m	35	30.47 ± 19.78	132.65 ± 26.20
	*Schizothorax pseudaksaiensis*	Jun. 2021 (Summer)	E80°56′1.68″, N42°57′22.6″	697 m	40	161.22 ± 97.81	212.23 ± 41.76
Bone sample	*A. laticeps*	Same as above	Same as above	Same as above	1	220.72	76.47
	*D. maculatus*				2	236.48 ± 10.75	69.76 ± 9.43
	*S. pseudaksaiensis*				2	270.00 ± 20.00	300.00 ± 50.00

**Table 2:** Morphological factors and morphological indices of the otoliths of three Schizothoracinae species.

Morphologic Factor	Morphologic Index
*F*_1_ = OP/(OA)^1/2^	Roundness (*R*o) = 4OA/ΠOL^2^
*F*_2_ = OP/OL	Format factor (*F*f) = 4ΠOA/OP^2^
*F*_3_ = OP/OW	Circularity (*C*) = OP^2^/OA
*F*_4_ = (OA)^1/2^/OL	Rectangularity (*R*e) = OA/(OL × OW)
*F*_5_ = (OA)^1/2^/OW	Ellipticity (*E*) = (OL + OW)/(OL − OW)
*F*_6_ = OW/OL	Radius ratio (*R*r) = R_max_/R_min_
	Aspect ratio (*A*r) = OL/OW

**Table 3:** Morphological parameters of the otoliths of three Schizothoracinae species (F = statistics, *p* = probability value).

Morphological Parameter	*A. laticeps*		*D. maculatus*		*S. pseudaksaiensis*		F	*p*
	Range	Mean ± S.D.	Range	Mean ± S.D.	Range	Mean ± S.D.		
OA/mm^2^	1.11~2.01	1.47 ± 0.24	0.61~1.99	1.29 ± 0.40	0.02~0.30	0.18 ± 0.07	321.406	0.000
R_min_/mm	1.19~2.31	1.66 ± 0.30	0.45~1.92	1.12 ± 0.41	0.03~0.23	0.11 ± 0.05	343.116	0.000
R_max_/mm	1.52~3.10	2.21 ± 0.44	0.60~2.55	1.51 ± 0.57	0.03~0.34	0.17 ± 0.08	297.452	0.000
OP/mm	4.55~9.12	6.56 ± 1.26	1.75~7.83	4.46 ± 1.66	0.10~0.98	0.46 ± 0.22	317.320	0.000
OL/mm	0.72~1.51	1.07 ± 0.22	0.30~1.25	0.73 ± 0.27	0.02~0.17	0.08 ± 0.04	289.738	0.000
OW/mm	0.61~1.16	0.84 ± 0.15	0.23~0.97	0.57 ± 0.21	0.01~0.12	0.06 ± 0.03	343.530	0.000

OA, otolith area; R_min_, minimum radius of otolith; R_max_, maximum radius of otolith; OP, otolith perimeter; OL, otolith length; OW, otolith width; F, statistics; *p*, probability value.

**Table 4:** The results of nonparametric ANOVA for 13 otolith morphological indices of three Schizothoracinae species.

Index	Min–Max	Kruskal-Wallis H (k)	F	*p*
*F*_1_	0.56–7.52	108.678	545.968	0.000
*F*_2_	4.79–8.22	22.109	12.551	0.000
*F*_3_	7.01–10.25	14.140	4.716	0.010
*F*_4_	0.91–10.38	107.201	301.479	0.000
*F*_5_	1.17–14.51	110.954	327.522	0.000
*F*_6_	0.60–0.87	38.000	32.602	0.000
*R*o	1.06–137.26	107.201	111.787	0.000
*F*f	0.22–39.49	108.678	124.625	0.000
*C*	0.32–56.49	108.678	268.631	0.000
*R*e	1.08–150.69	109.303	114.277	0.000
*E*	4.05–14.32	38.000	19.586	0.000
*R*r	1.23–1.66	57.406	65.022	0.000
*A*r	1.15–1.65	38.000	32.218	0.000

*F*_1_–*F*_6_, Six otolith morphological factors; *R*o, Roundness; *F*f, Format factor; *C*, Circularity; *R*e, Rectangularity; *E*, Ellipticity; *R*r, Radius ratio; *A*r, Aspect ratio; F, statistics; *p*, probability value.

**Table 5:** Results of discriminant analyses of three Schizothoracinae species.

	Species	Number	Identification Accuracy/%	Adjudicated Population		
				*A. laticeps*	*D. maculatus*	*S. pseudaksaiensis*
13 otolith morphological indicators	*A. laticeps*	63	93.70	59	4	0
	*D. maculatus*	35	88.60	4	31	0
	*S. pseudaksaiensis*	40	100.00	0	0	40
	Total	138	94.20			
77 Fourier harmonics	*A. laticeps*	63	100.00	63	0	0
	*D. maculatus*	35	100.00	0	35	0
	*S. pseudaksaiensis*	40	100.00	0	0	40
	Total	138	100.00			

**Table 6:** Comparison of the number of odd fin bones among the three Schizothoracinae species.

	*A. laticeps*			*D. maculatus*			*S. pseudaksaiensis*		
	Dorsal Fin	Anal Fin	Tail Fin	Dorsal Fin	Anal Fin	Tail Fin	Dorsal Fin	Anal Fin	Tail Fin
Number of fin spines	3	2	—	2	2	—	3	3	—
Number of fin rays	7	2	—	8	5	—	6	5	—
Number of branching fin rays	8	5	17	8	6	17	8	6	17

## Data Availability

The data that support the findings of this study are available from the corresponding author upon reasonable request.

## Appendix A

**Table A1:** Initial eigenvalues and contribution ratios of the first three principal components of the morphology indices of the three Schizothoracinae species.

Proportion of Character	Principle Component (PC)		
	1	2	3
*F*_1_	−2.78934	−1.23701	1.01004
*F*_2_	−1.57542	1.3931	6.16384
*F*_3_	1.15785	−1.87329	6.33086
*F*_4_	2.96144	1.65461	0.51855
*F*_5_	3.0626	1.26527	0.46233
*F*_6_	−2.40755	2.95002	0.13973
*R*o	2.78448	1.88109	0.97529
*F*f	2.87886	1.6159	0.55485
C	−2.50524	−1.30465	1.57406
*R*e	2.85282	1.65712	0.95278
E	−2.1319	3.11362	−0.16803
*R*r	2.55303	−1.96379	−0.06983
*A*r	2.45229	−2.85188	−0.27932
Principal component eigenvalue	8.063	2.588	1.418
Contribution ratio/%	62.022%	19.903%	10.905%
Cumulative contribution ratio/%	62.022%	81.928%	92.834%

**Table A2:** Principal component analysis of the otolith morphology of three Schizothoracinae species based on Fourier transform analysis.

Principal Component	Eigenvalues	Contribution Rate/%	Cumulative Contribution Rate/%
1	0.0038	34.8875	34.8875
2	0.0017	15.2342	50.1217
3	0.0013	11.4603	61.582
4	0.0008	7.5438	69.1258
5	0.0006	5.7237	74.8496
6	0.0005	4.3391	79.1887
7	0.0004	3.9285	83.1172
8	0.0003	2.3482	85.4654
9	0.0002	2.1896	87.655
10	0.0002	2.0448	89.6997
11	0.0002	1.6885	91.3882
12	0.0001	1.2546	92.6428
13	0.0001	0.9606	93.6034
14	0.0001	0.8508	94.4543
15	0.0001	0.6071	95.0613
16	0.0001	0.5443	95.6056
17	0.0001	0.4902	96.0958
18	0.0000	0.43	96.5258
19	0.0000	0.3564	96.8821
20	0.0000	0.3508	97.2329

**Table A3:** Euclidean distances of three Schizothoracinae species.

	Species	*A. laticeps*	*D. maculatus*	*S. pseudaksaiensis*
13 otolith morphological indicators	*A. laticeps*	0	205.802	5834.272
	*D. maculatus*	205.802	0	4793.572
	*S. pseudaksaiensis*	5834.272	4793.572	0
77 Fourier harmonics	*A. laticeps*	0	0.004	0.014
	*D. maculatus*	0.004	0	0.015
	*S. pseudaksaiensis*	0.014	0.015	0
